# Comparison of Azithromycin and Clarithromycin Triple Therapy Regimens for Helicobacter Pylori Eradication in Hemodialysis Patients

**DOI:** 10.5812/numonthly.2794

**Published:** 2012-06-20

**Authors:** Mojgan Jalalzadeh, Morteza Nazarian, Jamshid Vafaeimanesh, Fatemeh Mirzamohammadi

**Affiliations:** 1 Departments of Nephrology, Imam Hossein Hospital, Shahid Beheshti University of Medical Sciences, Tehran, IR Iran; 2 Departments of Gastroetrology, Vali-e-asr Hospital, Zanjan University of Medical Sciences, Zanjan, IR Iran; 3 Departments of Internal Medicine, Qom University of Medical Sciences, Qom, IR Iran; 4 Student Research Committee, Vali-e-asr Hospital, Zanjan University of Medical Sciences, Zanjan, IR Iran

**Keywords:** Azithromycin, Clarithromycin, Helicobacter Pylori

## Abstract

**Background:**

Helicobacter pylori eradication with clarithromycin is more expensive than with azithromycin.

**Objectives:**

This study aimed to compare the effectiveness of these two antibiotics in eradicating H. pylori in hemodialysis (HD) patients.

**Patients and Methods:**

This is a prospective, randomized, double-blinded clinical trial analysis of HD patients. Patients who had dyspepsia and showed positive results for two of three tests, anti-H. pylori serology, H. pylori stool antigen (HpSAg), or Urease Breath Test (UBT), were included in the study. The subjects consisted of 39 dialysis patients who were randomly divided into two groups that received medication twice daily. Group OAC received 20 mg omeprazol, 500 mg amoxycilin, and 250 mg clarithromycin, and Group OAAz received 20 mg omeprazol, 500 mg amoxicillin, and 250 mg azithromycin. Both regimens were administered for 14 days. Eradication was investigated by performing the UBT and the HpSAg test eight weeks later.

**Results:**

This study began with 39 patients, 37 of which completed the treatment schedule (20 males and 17 females, mean age 59 years). Two patients died due to MI before beginning treatment. In the OAC group, negative results on the UBT and HpSAg tests were found in 82.4% and 88.2% of the participants, respectively. In the OAAz group, these values were 80% and 85%, respectively. The data showed that the difference between the two regimens was not significant (P = 1.0).

**Conclusions:**

According to the data, no differences in eradication rates were apparent between the azitromycin and the claritromycin regimens. However, lower cost and fewer complaints could be considered as an advantage of the triple therapy with azithromycin.

## 1. Background

Helicobacter pylori is a gram-negative, non-invasive bacillus that is usually acquired in childhood and lives in the gastric mucus. Transmission occurs by the fecal-oral or the oral-oral route (1). H. pylori is easily cultured from vomitus and gastroesophageal refluxate, and less easily from the stool (2). H. pylori is a common human pathogen and plays a role in the development of gastrointestinal (GI) symptoms (3). H. pylori is considered as one of the causes of gastritis and gastric ulcer, and it was also associated with gastric adenocarcinoma and gastric lymphoma (4). Peptic ulcer disease occurs in up to 25% of patients with chronic kidney disease (CKD) (5). Hemodialysis (HD) patients complain of different GI symptoms, such as nausea, vomiting, epigastric pain, postprandial fullness, early satiety, bloating, and eructation (6). In dialysis patients, digestive tract hemorrhage is sometimes fatal (7). Hyper-gastrinaemia, secondary hyper-parathyroidism, medications, and H. pylori infection are suggested to be among the factors that are causally linked to peptic ulcer disease in CKD patients (5). There are many issues related to H. pylori infection in patients with end-stage renal disease (ESRD), particularly in patients undergoing long-term dialysis (7, 8).

The American College of Gastroenterology has recommended that all patients with ulcer who are infected with H. pylori should receive treatment to eradicate it (9). There is an abundant body of literature on various aspects of treatment regimens for H. pylori infections (10). It is now believed that triple or quadruple medication therapy should be administered for 10–14 days to help eradication (11, 12). A common regimen that does not rely on metronidazole includes clarithromycin, amoxicillin, and either omeprazole or lansoprazole (13). However, the rate of clarithromycin resistance is 7–10% in the US, and even higher in other countries (13-16), and this is why more effective regimens are being sought. Azithromycin, medication similar to clarithromycin, showed potential promises during preliminary testing (16). Azithromycin is an azalide similar to clarithromycin but less expensive and less prone to select for resistance (13). Most of the clinical trials examined short-duration azithromycin regimens in treating H. pylori (2–7 days of therapy), which may explain the range of eradication rates observed, from 44% to 93% (17-19).

## 2. Objectives

To determine the effectiveness of these regimens in HD patients, we compared 14-day azithromycin and clarithromycin therapies in eradicating H. pylori.

## 3. Patients and Methods

This prospective, randomized, double-blinded clinical trial was conducted between March 2008 and October 2008. Thirty-nine dialysis patients, aged 23–88 years, were enrolled in the study. They underwent dialysis at two HD centers in the Iranian provincial capital of Zanjan. Thirty-seven patients completed the treatment schedule (20 men and 19 women, with a mean age of 59 years). Two patients died due to MI and were excluded from the study before it started. The mean duration of dialysis was 40.26 ± 34.8 months. The patients gave informed consent before their initial evaluation for upper GI tract symptoms. Those who had dyspepsia and were diagnosed with H. pylori on initial testing were included in the study. To confirm H. pylori infection, three types of tests were used: anti H. pylori serology (based on an ELISA test), H. pylori stool Ag (HpSA, based on ELISA polyclonal antibodies Premier Platinum), and the urease breath test (UBT, based on the modified European protocol). If two of these tests were positive, the patients were considered infected.

Subsequently, participants were administered two weeks of triple therapy. Eight weeks after completing therapy, the stool antigen and UB tests were performed. The exclusion criteria included a history of previous H. pylori treatment, the use of any of the proposed antibiotics over the previous six months, any known allergy to those medications, as well as patients’ non-cooperation in undergoing the UBT. Patients were randomly assigned to one of two treatments groups. The OAC group received a regimen containing 20 mg omeprazole, 500 mg amoxicillin, and 250 mg clarithromycin twice a day for 14 days. The OAAz group received a regimen containing 20 mg omeprazole, 500 mg amoxicillin, and 250 mg azithromycin, also administered twice a day for 14 days. Meantime, we continued the administration of omeprazol to both groups for two additional weeks. During the study, all patients were administered recombinant erythropoietin (rEPO) at a dose of 360 U/kg/wk. The subjects did not know what medications they were receiving. We assigned a nurse in the ward to administer the medications, which were placed in unidentifiable envelopes. The attending physicians were also unaware of patient/group categories. At the end of the study, our colleague in charge of analyzing the data received the information related to the type of pills administered to the patients by the nurse.

The subjects were followed for a total of 10 weeks, which included the treatment period. Any side effects and symptom changes were recorded. Exit interviews and pill counts were conducted to evaluate compliance and to determine side effects. After the 10-week period, patients were re-evaluated for H. pylori infection by UBT and HpSAg. The evaluation for eradication included negative results on the UBT and the HpSAg tests. The Zanjan University of Medical Sciences ethics committee for research approved the study (Ethics No; 19/3-304488). Trial Registration Number: IRCT138811223325N1.

### 3.1. Statistics

A Chi-square test with Pearson’s correction was done to compare string variables between groups. A Student’s independent T-test for quantitative variables with normal distribution and a Mann-Whitney U-test for numeric variables without normal distribution were conducted. The paired t-test was used to compare variables before and after H. pylori eradication in the OAAz patient group. All statistical tests were two sided. P values of less than 0.05 were considered significant.

## 4. Results

Out of the 98 HD patients referred to the dialysis centers of the Zanjan University hospitals, 39 patients with a history of dyspepsia and positive results at two of three H. pylori diagnostic tests – anti H. pylori serology, HpSAg, and UBT, were enrolled into this study (Figure). Patients were randomly divided into the OAAz (n = 20) and OAC (n = 19) groups. Two patients in the OAC group died before treatment started, and were excluded from the study. Eleven patients (55%) in the OAAz group and eight patients (42%) in the OAC group were female, while nine patients (45%) in the OAAz group and 11 patients (57%) in the OAC group were male. The mean age of the patients in the two groups was 59 years. Demographic features of the patients in the two groups are shown in Table 1. Accordingly, no significant statistical differences were observed between the two groups in terms of gender, age, duration of dialysis, H. pylori infection, and history of dyspepsia.

**Figure:**
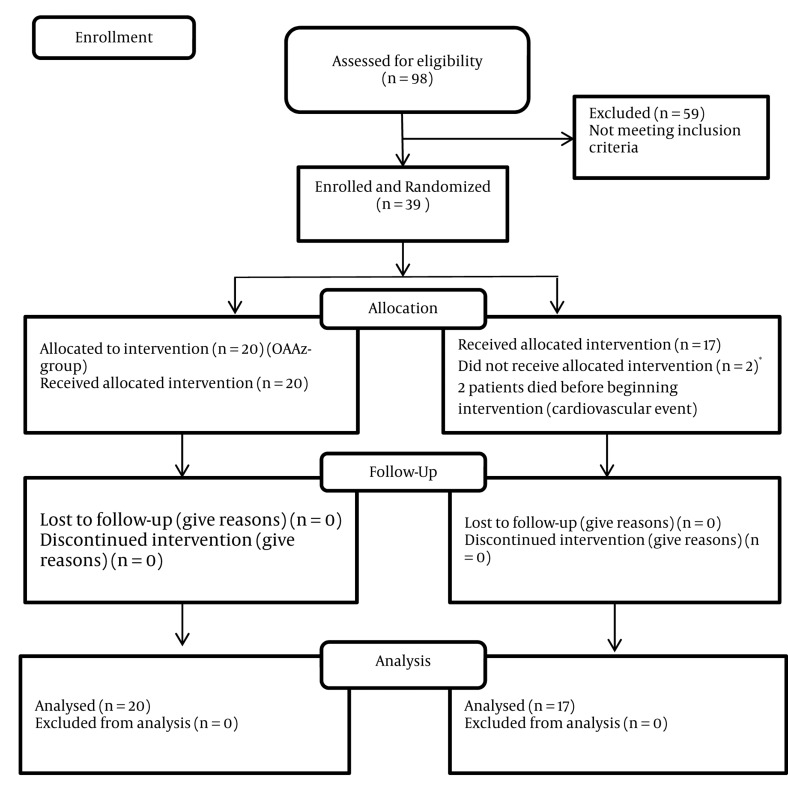
Consort 2008 Flow Diagram

**Table 1:** Comparison of the Demographic and Laboratory Variables in the Two Groups Before Treatment

Variables\Groups	OAAz ^a^ (n = 20)	OAC ^a^ (n = 17)	P value
Gender, male	9 (45%)	11 (57.9%)	0.52
Mean age, y	59.00	59.05	0.99
Duration of dialysis, mon	41.6	48.8	0.80
Cr ^a^, mg/dL	9 ± 2.9	10.59 ± 3	0.1
BUN ^a^, mg/dL	77.82 ± 33	99.10 ± 48	0.1
Alb ^a^, mg/dL	4.15 ± 0.6	4.20 ± 0.5	0.79
TG ^a^, mg/dL	148 ± 75	154 ± 82	0.83
Chol ^a^, mg/dL	172 ± 42	185 ± 45	0.36
Hgb ^a^, g/dL	10.3 ± 2	10.9 ± 2.1	0.40
EPO dosage, unit/wk	9133	8533	0.6
Ca ^a^, mg/dL	9.5 ± 1.3	9.5 ± 0.8	0.9
P ^a^, mg/dL	5.6 ± 1.5	5.9 ± 1.9	0.69
Kt/v ^a, b^	1.25 ± 0.33	1.06 ± 0.31	0.20
BMI ^a^, kg/m^2^	24.4 ± 3.4	24.8 ± 4.1	0.80

^a^Abbreviations: Alb, serum albumin; BMI, body mass index; BUN, blood urea nitrogen; Ca, calcium; Chol, cholesterol; Cr, creatinine; Hgb, hemoglobin; kt/v, “K” stands for clearance; “t” for time and “v” for volume; OAAz, omeprazole, amoxicillin, and azithromycin; OAC, omeprazole, amoxicillin, and clarithromycin; P, phosphor; TG, triglyceride

^b^Kt/v is the equation used for evaluating dialysis quality

Before and after triple therapy, blood samples were collected to measure the serum creatinine (Cr), albumin (Alb), triglyceride (TG), cholesterol (Chol), calcium (Ca), blood urea nitrogen (BUN), and hemoglobin (Hgb) levels. As shown in Table 1 and Table 2, no statistical differences were found between the two groups in the above-mentioned variables before triple therapy began. However, the mean Cr level in the OAC group was significantly higher than in the OAAz group after triple therapy (P = 0.05). In this study, a significant decrease in serum albumin levels was observed in the OAAz group (P = 0.009). Also, hemoglobin significantly increased in the OAAz group (P = 0.02). However, no significant differences were noted in the serum Cr, BUN, TG, Chol, and Ca levels in this group (Table 3). The average cost of anti-H. pylori therapy is $17.05, while the average cost of OAAz therapy is $5.60. This difference is statistically significant (P = 0.009).

**Table 2:** Comparison of the Variables in the Two Groups After Treatment (Independent Samples T-test)

Variables\Groups	OAAz (n = 20)	OAC (n = 17)	P value
Cr ^a^	8.6	10.4	0.05
BUN ^a^	67.5	66.3	0.83
Alb ^a^	3.8 ± 0.56	3.7 ± 0.51	0.46
TG ^a^	135 ± 71	145 ± 83	0.68
Chol ^a^	162 ± 41	159 ± 32	0.82
Hgb ^a^	11.4 ± 1.5	11.7 ± 1.8	0.65
rEPO ^a^ dosage	8400	8340	0.71
Ca ^a^	9.2 ± 1	9.1 ± 0.8	0.67
P ^a^	5.8 ± 1.9	5.6 ± 1.3	0.77
Kt/v ^a,b^	1.29 ± 0.39	1.03 ± 0.33	0.17

^a^Abbreviations: Alb, serum albumin; BUN, blood urea nitrogen; Ca, calcium; Chol, cholesterol; Cr, creatinine; Hgb, hemoglobin; kt/v, “K” stands for clearance; “t” for time and “v” for volume; P, phosphorus; TG, triglyceride

^b^Kt/v is the equation used for evaluation dialysis quality

In the OAC group, after H. pylori eradication, a statistically significant reduction was observed in the serum BUN (P = 0.01), albumin (P = 0.01), cholesterol (P = 0.003), and calcium (P = 0.03) levels. However, in contrast to the OAAz group, the hemoglobin level did not increase in the OAC group (Table 3).

**Table 3:** Comparing the Variables in the Two Groups Before and After Treatment (Paired T-test)

OAAz ^a^ (n = 20)	OAC ^a^ (n = 17)	P value	OAAz (n = 20)
Gender, male	9 (45%)	11 (57.9%)	0.52
Mean age, y	59.00	59.05	0.99
Duration of dialysis, mo	41.6	48.8	0.80
Cr ^a^, mg/dL	9 ± 2.9	10.59 ± 3	0.1
BUN ^a^, mg/dL	77.82 ± 33	99.10 ± 48	0.1
Alb^a^, mg/dL	4.15 ± 0.6	4.20 ± 0.5	0.79
TG ^a^, mg/dL	148 ± 75	154 ± 82	0.83
Chol ^a^, mg/dL	172 ± 42	185 ± 45	0.36
Hgb ^a^, g/dL	10.3 ± 2	10.9 ± 2.1	0.40
EPO dosage, unit/Wk	9133	8533	0.6
Ca ^a^ (mg/dl)	9.5 ± 1.3	9.5 ± 0.8	0.9
P ^a^, mg/dL	5.6 ± 1.5	5.9 ± 1.9	0.69
Kt/v^a,b^	1.25 ± 0.33	1.06 ± 0.31	0.20
BMI ^a,^ kg/m2	24.4 ± 3.4	24.8 ± 4.1	0.80

^a^Abbreviations: Alb, serum albumin; BMI, body mass index; BUN, blood urea nitrogen; Ca, calcium; Chol, cholesterol; Cr, creatinine; EPO, erythropoietin; Hgb, hemoglobin; kt/v, “K” stands for clearance; “t” for time and “v” for volume; OAAz, omeprazole, amoxicillin, and azithromycin; OAC, omeprazole, amoxicillin, and clarithromycin; P, phosphorus; TG, triglyceride

^b^Kt/v is the equation used for evaluation dialysis quality

### 4.1. Efficacy of Triple Therapy in H. pylori Eradication in the Two Groups

After eight weeks of treatment, H. pylori eradication was assessed by UBT and HpSAg. Sixteen patients (80%) in the OAAz group and 14 patients (82.4%) in the OAC group were negative by the UBT, while 17 patients (85%) in the OAAz group and 15 patients (88.2%) in the OAC group were negative by the HpSAg test. No statistically significant differences in the eradication rates were observed between the two study groups (Table 4).

**Table 4:** H. pylori Eradication Efficacy of Triple Therapy in Hemodialysis Patients Tests\Groups

Variables/Groups	OAAz^a^ (n = 20)	OAC ^a^ (n = 17)	P value
Cr ^a^, mg/dL	8.6	10.4	0.05
BUN ^a^, mg/dL	67.5	66.3	0.83
Alb ^a^, mg/dL	3.8 ± 0.56	3.7 ± 0.51	0.46
TG ^a^, mg/dL	135 ± 71	145 ± 83	0.68
Chol ^a^, mg/dL	162 ± 41	159 ± 32	0.82
Hgb ^a^, g/dL	11.4 ± 1.5	11.7 ± 1.8	0.65
rEPO ^a^ dosage	8400	8340	0.71
Ca ^a^, mg/dL	9.2 ± 1	9.1 ± 0.8	0.67
P ^a^, mg/dL	5.8 ± 1.9	5.6 ± 1.3	0.77
Kt/v ^a,b^	1.29 ± 0.39	1.03 ± 0.33	0.17
BMI ^a^, kg/m2	24.9 ± 3.5	25.7 ± 4.3	0.69
Drug costs for a patient (Dolor)	5.60	17.05	0.009

^a^Abbreviations: Alb, serum albumin; BMI, body mass index; BUN, blood urea nitrogen; Ca, calcium; Chol, cholesterol; Cr, creatinine; rEPO, recombinant erythropoietin; Hgb, hemoglobin; kt/v, “K” stands for clearance; “t” for time and “v” for volume; OAAz, omeprazole, amoxicillin, and azithromycin; OAC, omeprazole, amoxicillin, and clarithromycin; P, phosphorus; TG, triglyceride;

^b^Kt/v is the equation used for evaluation dialysis quality

## 5. Discussion

H. pylori has high urease activity that produces ammonia in the presence of urea. In patients with ESRD, blood urea levels and gastric juice urea nitrogen levels are high (20). One of the etiological factors involved in gastric mucosal disorders is thought to be ammonia (21). Several researchers have suggested that a higher concentration of urea in the gastric juice of CKD patients would raise the local gastric pH and thus provide enough substrate for H. pylori growth (22). Other investigators obtained opposite results (23, 24).

Few studies have focused on the relationship between H. pylori infection and the duration of dialysis (1, 7). They found that the proportion of H. pylori-positive patients was significantly lower in patients receiving dialysis for longer periods (1, 23, 25). Studies have shown that multiple drug regimens are beneficial for eradicating H. pylori. Antibiotics used for various regimens comprise azithromycin, clarithromycin, amoxicicilin, tetracycline, levofloxacin, furazolidone, rifabutin, and metronidazole (16). We performed a randomized, blinded comparison between 14-day triple therapy regimens in which azithromycin was changed to clarithromycin, for H. pylori eradication in patients with ESRD, who were treated for HD. We achieved a high rate of H. pylori eradication (81%) in both groups, with no statistical differences between the two regimens. However, we recommend a longer follow-up study to help assess the long-term implications of this regimen.

Four previous trials evaluated azithromycin-based regimens. Al-Assi et al. used azithromycin, tetracycline, and bismuth, and found a 50% eradication rate (26). Another study by Vcev et al. found an eradication rate of 85% with omeprazole, azithromycin, and amoxicillin (19). A third study, also by Vcev et al., examined the effectiveness of pantoprazole and amoxicillin with either azithromycin or clarithromycin. The eradication rates in this study were found to be 71% and 81%, respectively (19). A fourth study, by Laurent et al., using azithromycin, amoxicillin, and omeprazole, had an eradication rate of 38% (27). In vitro, azithromycin is bactericidal against H. pylori. In 1998, a study revealed that azithromycin becomes concentrated in the gastric mucosa in much higher levels than in the plasma (17). The finding supported the hypothesis that azithromycin is an effective antibiotic for treating H. pylori infections (28). However, in 1999, it was found that although the antibiotic is delivered to the appropriate target organ, it does not seem to reach the desired concentration in the gastric fluid and in the mucous layer where the organism lives (29). This most likely explains the inability of azithromycin to achieve appropriate eradication rates (29).

Other studies showed that both azithromycin and clarithromycin achieve high extracellular concentrations (30). However, the shorter half-life of clarithromycin may increase the dissemination of H. pylori-resistant organisms into the community (31). There is also a possibility that H. pylori has nutritional effects (32). Our study showed that after H. pylori eradication in both groups, serum albumin levels decreased significantly. The study also showed that in the claritromycin group, the levels of serum cholesterol, BUN, and calcium decreased significantly after treatment as compared to the azithromycin group. One study showed that the non-uremic H. pylori infected group had a significantly greater frequency of hypo-proteinemia as compared with the H. pylori-negative group (33), and significant increases were recorded in the body weight and in the serum levels of total cholesterol, total proteins, and albumin after H. pylori eradication.

Furthermore, malnutrition is common among patients with chronic renal failure. Studies about the relation of H. pylori infection with malnutrition in a HD population revealed contradictory results. Infection with H. pylori was shown to be associated with anorexia, inflammation, and malnutrition in dialysis patients (34). However, other studies reported opposite effects (35-37). Seung Ha Park and colleagues found that H. pylori eradication had no effect on metabolic and inflammatory parameters, including blood sugar, lipid profiles, insulin resistance, white blood cell count, and C-reactive protein (38). In our study, the mean serum Alb level eight weeks following H. pylori eradication decreased significantly from 4.2 to 3.6 (P < 0.001). Contrary to the aforementioned studies, which suggest that H. pylori eradication leads to positive metabolic changes, our study did not show this. We also found that in the OAC group, after eradication of H. pylori, there was a significant decrease in the level of serum cholesterol, BUN, and calcium. We do not have an explanation for this.

Each of these two antibiotics has some side effects. For clarithromycin, the most common ones are a metallic (bitter) taste, loss of appetite, nausea, vomiting, and diarrhea (37). Azithromycin is generally well tolerated, but its most common side effects are diarrhea or loose stools, nausea, abdominal pain, and vomiting, which may occur in fewer than one in twenty patients receiving the antibiotic (39). GI side effects have been reported in 10–20% of clarithromycin-treated patients and in 10% of the azithromycin-treated patients (40); these results are in conformity with the results of our study. Even though the patients from the clarithromycin group had more GI complaints, the complaints were not severe enough to compel the patients to drop out of the study. In addition, in the azithromycin group, hemoglobin levels increased more than in the claritromycin group, even though all the patients were administered rEPO at a 360 U/kg/wk dose. This increased hemoglobin level may be considered as another advantage of azitromycin over claritromycin, in addition to the low cost and better tolerance. However, despite these advantages, more studies are needed to compare and understand the effects of these medications on eradicating H. pylori infections in HD patients.
